# Rb and hematopoiesis: stem cells to anemia

**DOI:** 10.1186/1747-1028-3-13

**Published:** 2008-09-08

**Authors:** Carl R Walkley, Vijay G Sankaran, Stuart H Orkin

**Affiliations:** 1 Department of Pediatric Oncology, Dana-Farber Cancer Institute, Division of Hematology/Oncology and Stem Cell Program, Children's Hospital Boston, Harvard Stem Cell Institute, Harvard Medical School, Boston, MA, 02115, USA; 2 Howard Hughes Medical Institute, Boston, MA, 02115, USA; 3 St. Vincent's Institute, Department of Medicine at St. Vincent's Hospital, University of Melbourne, Fitzroy, VIC 3065, Australia

## Abstract

The retinoblastoma protein, Rb, was one of the first tumor suppressor genes identified as a result of the familial syndrome retinoblastoma. In the period since its identification and cloning a large number of studies have described its role in various cellular processes. The application of conditional somatic mutation with lineage and temporally controlled gene deletion strategies, thus circumventing the lethality associated with germ-line deletion of Rb, have allowed for a reanalysis of the *in vivo *role of Rb. In the hematopoietic system, such approaches have led to new insights into stem cell biology and the role of the microenvironment in regulating hematopoietic stem cell fate. They have also clarified the role that Rb plays during erythropoiesis and defined a novel mechanism linking mitochondrial function to terminal cell cycle withdrawal. These studies have shed light on the *in vivo *role of Rb in the regulation of hematopoiesis and also prompt further analysis of the role that Rb plays in both the regulation of hematopoietic stem cells and the terminal differentiation of their progeny.

## Introduction

Decisions to enter the cell cycle are regulated by the G_1_-S phase restriction point [1]. One of the major molecular circuits involved in this restriction point is centered on the retinoblastoma protein (Rb) and is termed the "Rb pathway" [2]. Through a series of sequential inhibitory phosphorylation events by cyclin dependent kinases (Cdk), Rb family members are inactivated and release E2F proteins which drive the cell through the G_1 _phase into S phase where cell division will occur independent of extracellular signals [3,4]. Mutations of Rb itself and other components of the "Rb pathway" occur universally in human cancer, where differentiation is perturbed, and cancer initiating cells are thought to reacquire the capacity to self-renew [5,6]. Rb has also been linked to the cell cycle arrest that accompanies terminal differentiation. As such the study of the role of these pathways in self-renewal will improve our understanding not only of the normal regulation of self-renewal but will also be applicable to understanding the initiation and maintenance of cancer.

Hematopoiesis represents an attractive system in which to study the role of the cell cycle in the regulation of cell fate. Hematopoiesis is hierachically structured, defined cell populations can be prospectively isolated and manipulated using flow cytometry and rigorous analysis of stem cell and lineage restricted progenitor function can be performed using transplantation assays. Coupling of the advances in *in vivo *genetic manipulation techniques, such as Cre-lox technologies, with the study of hematopoiesis and HSCs has led to rapid advances in our understanding of the programs involved in the maintenance of HSC self-renewal and differentiation and in the identification of transcription factors critical for lineage choice and identity [7-13]. Recent studies have sought to define the role that the cell cycle control machinery plays in the regulation of HSC fate and in the differentiation of specific hematopoietic lineages such as neutrophils and erythroid cells [14-17]. These studies, particularly those focused on the retinoblastoma protein (Rb), have revealed new roles for the cell cycle in the regulation of hematopoietic stem cells and in the coupling of cell cycle arrest to cellular differentiation. This review will focus on the role of Rb in HSC biology and in the terminal differentiation of the erythroid lineage.

### A historical perspective on the role of Rb in hematopoiesis

Beginning with the initial descriptions of the germline knockout mice for the Rb gene in 1992, a role for Rb in hematopoiesis, and in particular erythropoiesis, was suspected. Rb deficient embyros had a profound anemia, proposed to lead to embryonic lethality, along with marked neurodevelopmental defects [18-20]. However upon further examination of chimeric mice it was noted that Rb-null cells could contribute to hematopoiesis and generate erythroid cells [21,22]. Hematopoietic contribution in the chimeric animals was largely normal. These observations raised the possibility that the anemia in the Rb-null embryos resulted from non-cell autonomous defects.

The nature of the influence of other cell types, hematopoietic or otherwise, to the described phenotypes remained unclear and furthermore it was uncertain whether Rb had any intrinsic role in erythropoiesis. The former question was explored using several different approaches. One group found that the presence of a wild-type placenta in Rb-null embryos allowed their survival through the time of birth, although these animals did not appear to have completely normal development [23,24]. Using *in vitro *culture approaches conflicting results regarding the requirement for Rb became apparent. Rb was found to be an intrinsic role in limiting the proliferative capacity of erythroid progenitors during terminal erythroid differentiation [25], or, alternatively, to be necessary in macrophages to properly support erythroid cells that develop in a niche interaction involving these two cell types, termed the "erythroid island" [26,27]. More recent chimeric analysis suggested that Rb was necessary for *in vivo *stress responses and found a variety of variable phenotypes over time in hematopoiesis of chimeric animals [28]. Unfortunately, this work was limited because of the inability to distinguish cell-autonomous defects from a role in other cell types (such as macrophages) [28]. Additionally, some of this phenotypic variation may represent the presence of concomitant deletion of Rb in myeloid and niche cells causing a myeloproliferative-type disorder, as has been described [29].

The recent generation of conditional alleles of Rb and the application of Cre-lox technology has allow the development of complex *in vivo *models and a reanalysis of the role of Rb in a variety of biological processes. Utilizing conditional strategies, we and others have investigated the role that Rb plays in the regulation of hematopoietic stem cell biology and more recently sought to clarify the requirement for Rb during erythropoiesis. These models have revealed new insight not only into the biology of Rb and its role in hematopoiesis but also more broadly into the fundamental underlying processes that regulate hematopoietic stem cells and into the coupling of cell cycle exit to terminal differentiation.

### Cell cycle regulation and hematopoietic stem cells

The continual production of blood cells is maintained by a small number of stem cells (HSCs), which reside in a specialized microenvironment in the adult BM, termed the niche [30-32]. The niche is a complex three dimensional system comprising cellular, extracellular and mineral components [33]. It is within the niche that HSCs divide and undertake cell fate decisions to constantly replenish hematopoiesis through the processes of differentiation, ultimately producing mature blood cells, and self-renewal which results in the production of more HSCs to replenish and maintain the HSC pool throughout life. The fine regulation of HSCs involves cell division coupled with appropriate intrinsic and extrinsic cues, the latter principally derived from the niche environment.

In steady state conditions HSCs are in a slowly dividing state, termed relative quiescence, with a cell division cycle in the mouse in the range of 2–4 wks [34,35]. This is in contrast to the rapidly cycling hematopoietic progenitor cells, which are more committed to differentiation than HSCs. Engraftment of transplanted HSCs has been shown to be dependent on cell cycle status, with only cells in the non-cycling G_0_/G_1 _phase efficiently engrafting following transplantation and contributing to stable long-term hematopoiesis [36-39]. The slow cycling of HSCs acts to spare them from acute toxicity (such as chemotherapy), but may also prevent eradication of neoplastic cells [40,41]. In part, the dramatic contrast in cell cycle status between stem and progenitor cells has led to the hypothesis that cell cycle regulation plays a fundamentally important role in stem cell fate determination. This hypothesis is supported by recent data demonstrating a slower rate of division in Hoxb4^hi^Pbx1^lo ^cells, which extensively self-renewal *in vitro*, compared to control cells [42]. It is essential for an HSC to undergo cell division if it is to self-renew, but how the cell division cycle is integrated into the process of self-renewal is unclear. It is also unknown as to whether cell cycle regulation represents an intrinsic or extrinsic modifier of HSC fate.

Negative regulators of both Cdk2 and Cdk4/6 activity, and therefore Rb function, have been demonstrated to have roles in regulating HSCs [43-47]. For the most part however these phenotypes have been relatively subtle, particularly when compared to hematopoietic phenotypes apparent after disruption of transcription factors such as C/EBPα [10] and Tel [9] amongst others, and are often apparent only after serial transplantation. The "Rb pathway" has also been implicated in phenotypes observed in both the Bmi1^-/- ^and ATM^-/- ^HSCs [48-50]. Rb was also described as being an important regulator of stem cell maintenance in the plant species *Arabidopsis *[51]. The interaction of cell cycle regulators with other factors such as Hoxb4 or telomerase deficiency has produced much more striking phenotypes than that observed for the cell cycle mutants in isolation [52,53]. While clearly demonstrating that cell cycle modifiers have roles in regulating stem cells, particularly HSCs, the aforementioned studies have not been able to clearly discriminate between intrinsic or extrinsic contributions to HSC fate as all studies to date had utilized non-hematopoietic restricted mutant alleles. A recent study demonstrating that the *p27*^*Kip1-/- *^microenvironment mediates the myelo-lymphoid expansion observed in the *p27*^*Kip1-/- *^animals raises the possibility that the HSC expansion observed in *p27*^*Kip1-/- *^BM is extrinsic in nature [47,54]. This result suggested that cell cycle regulators may play a role in regulating the competence of the hematopoietic niche, in addition to having potential intrinsic roles in HSC fate determination.

### The hematopoietic stem cell niche

Recent studies have begun to characterize the nature of the adult BM niche [30-32,55-57]. Two major cell types have been identified as being important components of the HSC niche, the bone-forming osteoblast and the blood vessel lining endothelial cell, although there is still debate as to the extent of the contribution of each of these cell types to the HSC niche. Studies have shown that extrinsic regulation of hematopoiesis and HSCs can occur via modulation of osteoblast number and function [56-58]. Endothelial cells have also recently been suggested to play a critical role as part of the HSC niche [55,59]. A recent study suggested a common anatomical location for both osteoblast and endothelial cell types with respect to the niche, raising the possibility that they may collectively contribute to the function of the HSC niche [60]. Irrespective of the exact cellular composition of the niche, products of each of these cell types have been shown to have the potential to modulate HSC function. Additionally studies have shown that extrinsic regulation of homeostatic HSC numbers can be dominant to even very profound intrinsic cues *in vivo *[61,62]. It is therefore of major importance to further understand the roles of the different cell types comprising the HSC niche (osteoblast, endothelial cell) and delineate their effects on HSC fate decisions. This includes defining the molecular regulators of the niche cells and exploring regulatory interactions between the hematopoietic cells and the non-hematopoietic derived microenvironment. Despite the recent advances, little is known about the molecular regulators of hematopoietic niche competence or the involvement of the niche in the initiation and maintenance of hematopoietic diseases.

### A role for Rb in hematopoietic stem cell fate regulation

As a result of the embryonic lethality of Rb-deficient animals, somatic conditional inactivation or lineage restricted deletion of Rb is necessary to define its role in HSC fate. To enable analysis of the role of Rb specifically in HSCs, Rb^fl/fl ^animals [63,64] were crossed to the interferon inducible *Mx1*-Cre transgene [65]. Inducible somatic deletion in the adult has many advantages over non-inducible systems in the context of the analysis of HSCs, in particular the ability to transplant HSCs prior to gene inactivation which will restrict deletion to only the hematopoietic system. Using this approach we observed that HSC contribution to hematopoiesis was largely normal in the absence of Rb when the HSCs were supported by a wild-type microenvironment [66]. This result is consistent with that observed in chimeric Rb animals where hematopoietic development is essentially normal and Rb-deficient cells are capable of widespread contribution [21].

Whilst hematopoiesis was largely normal when Rb deficient cells were supported by a wild-type microenvironment a distinctly different phenotype was observed when *Mx1*-Cre Rb^fl/fl ^animals were induced to delete Rb [29]. This experimental design does not restrict gene deletion to the hematopoietic cells, but also results in gene deletion in the hematopoietic microenvironment [56] and other organs of the animal [65]. These animals rapidly developed myeloproliferation, with a dramatic expansion of the numbers of neutrophils in the bone marrow and extramedullary erythro- and myelopoiesis in the spleen. Stem and primitive progenitor cells were also mobilized to the periphery and were found in the spleen and peripheral blood. This myeloproliferation was stable and present for the lifespan of the animals, which in our care was approximately 8 months. At this time the animals present with a phenotype consistent with hematopoietic failure with a hypocellular bone marrow filled with mature neutrophils and a drastic reduction in splenic hematopoiesis, however the animals also developed pituitary tumors. This striking and profound phenotype was not present in a wild-type microenvironment.

When bone marrow HSC frequency was determined in these animals it was found to be reduced 5 fold with a concomitant increase at extramedullary sites, suggesting an overall redistribution of cells away from the bone marrow environment. The HSCs obtained from the bone marrow were able to reconstitute hematopoiesis in wild-type recipients and could be serially transplanted suggesting that when supported by a wild-type microenvironment the HSC itself was able to self-renew and differentiate relatively normally. Whilst the HSCs were functionally normal they were failing to be retained in the bone marrow microenvironment, placing Rb as an extrinsic regulator of HSC fate. These studies were supportive of the interpretation that stem cell self-renewal can occur independently of Rb, consistent with that reported from the analysis of embryonic stem cell self-renewal and differentiative cell cycles [67,68]. This observation is intriguing in light of the reacquisition of self-renewal potential during tumorigenesis. Human tumors are thought to near universally inactivate the "Rb pathway", and whilst it can not be assumed that the consequences of the mutations of various components of this pathway are equivalent, it does raise the possibility that inactivation of this pathway may facilitate reacquisition of the self-renewal program [5].

The failure to recapitulate myeloproliferation from Rb-deficient hematopoietic cells in a wild-type microenvironment implied a role for the microenvironment in its development. An Rb-deficient microenvironment did not cause myeloproliferation of wild-type hematopoietic cells, contrasting with that observed in the case of an RARγ-deficient microenvironment which was the sole cause of myeloproliferation in this model [69]. In Rb-deficient myeloproliferative animals a significant increase in ostecoclast number could be observed which correlated with a rapid loss of bone architecture and trabecular volume, both factors that have been implicated in the regulation of HSCs [56,57,70]. Based on these findings, we sought to determine if myeloid cells were required for the development of the myeloproliferation. Deletion of Rb from myeloid cells using *Lysozyme-M*-Cre did not result in myeloproliferation, but when combined with an Rb-deficient microenvironment a fatal myeloproliferation rapidly ensued [29]. This result demonstrated that interactions between hematopoietic cells and non-hematopoietic stromal elements could result in the development of myeloproliferation and additionally modulate HSC fate within the bone marrow microenvironment. The nature of the non-hematopoietic cell or cells responsible for this interaction are currently under investigation. Interestingly, studies using *Vav*-Cre to delete Rb reported a similar phenotype, although not as severe, to that we had observed with the *Mx1*-Cre based deletion of Rb [71]. *Vav*-Cre is known excise in both hematopoietic and vascular lineages, which directs attention to the role of the vasculature in the myeloproliferative phenotypes that were observed [72-74].

Several differences are observed between the data derived from *Vav*-Cre and *Mx1*-Cre mediated deletion of Rb in hematopoietic cells and HSCs. Daria *et al *observed a requirement for Rb in the stress response of HSCs and this has also previously been suggested in the context of the role of Rb in erythropoiesis [28,75]. The timing of gene deletion is also relevant for interpreting the phenotypes observed in these two models. Whilst with *Mx1*-Cre, gene deletion is largely temporally controlled and can be restricted to the adult HSC, *Vav*-Cre is active from the genesis of HSCs. *Mx1*-Cre could also be restricted to the HSC and subsequent hematopoiesis through transplantation prior to deletion of Rb where as deletion of Rb with *Vav *occurs in utero in both HSCs and vasculature potentially disturbing the microenvironment in which the HSCs reside and expand during development prior to the shift in hematopoiesis to the intramedullary sites of bone [76,77]. Also of note is that the cell division dynamics of HSCs change during development, from rapidly cycling and dividing cells during the fetal liver and early stages of life to relatively quiescent and more slowly cycling in the adult context [34,35,38,78,79]. Thus the role for Rb may be context dependent, both in terms of stress response and developmentally in the regulation of HSC fate.

### Red blood cells, anemia and Rb

#### Cell cycle regulation in erythropoiesis

Each day an average adult human produces nearly 200 billion RBCs. To maintain the effective production of RBCs, a rapid proliferative expansion of early progenitors needs to occur. This expansion is followed by termination of proliferation and commencement of the complex biogenic program allowing production of the hemoglobin necessary for the oxygen-carrying capability of the RBCs. If any step in this process is disrupted, as occurs in numerous human diseases, then anemia results [80]. Modulation of erythroid cell cycle regulation has been exploited therapeutically, particularly in patients with diseases due to defects in hemoglobin structure or production, but more efficacious therapies will depend on an increased understanding of this process [81-83].

The variation in cellular proliferation during erythropoiesis has been well-studied at a descriptive level over many decades and the stages at which alterations in this process occur have been characterized [80,84-86]. However, an in-depth understanding of the molecular control of this process is largely lacking. It is known that early bipotential megakaryocyte-erythroid progenitors (MEPs) and early erythroid progenitors (BFU-Es) proliferate at a relatively slow rate, but that this level of cell cycle progression is necessary to maintain the pool of more differentiated precursors. This has been demonstrated through the study of mutations that disrupt the activity of the c-Myb gene, suggesting a critical role for cell cycle regulation at these early stages of differentiation [87-89].

Following these early stages, a rapid rise in proliferation is observed at the colony-forming unit erythroid (CFU-E) stage of differentiation. After the CFU-E stage, the erythroid progenitors undergo three to five additional cell divisions whilst maturing into erythroblasts that then need to undergo terminal cell cycle exit at the G1 phase to facilitate complete differentiation. Ultimately the post-mitotic cells undergo further maturation and eventually enucleate to give rise to the functional RBCs that can enter the circulation and play a critical role in oxygen transport. Our understanding of how cell cycle exit is carried out during erythroblast maturation and how this is coupled to differentiation is limited. Studies in erythroid cell lines have suggested some molecular players involved in this process [90], but our understanding of the control of this process *in vivo *is incomplete. Since it is known that G1 exit frequently requires the activity of Rb to occur in other cell types [63,91], it is important to understand the role that this gene plays in the process of erythropoiesis.

#### The role of Rb in erythropoiesis

In order to directly examine whether Rb had any intrinsic or potentially extrinsic role in erythropoiesis, we utilized conditional deletion of the gene in a variety of hematopoietic lineages [92]. We were able to delete Rb specifically within the erythroid lineage using the erythropoietin receptor knock-in GFPcre mouse line (*EpoR*-GFPcre) [93,94]. We could also delete Rb within the macrophage and granulocyte lineage using *Lysozyme-M*-Cre [95] and somatically within the entire adult hematopoietic system using *Mx1*-Cre [65]. The results of this analysis indicated that Rb was necessary within the erythroid cells for normal erythropoiesis, but was dispensible in macrophages. This finding suggests that the use of *in vitro *reconstituted erythroid islands may not faithfully mimic physiological situations with the culture conditions used in these experiments [27]. Alternatively, it is possible that there may be compensation for the loss of Rb in macrophages *in vivo*.

Mice harboring an Rb deletion within the erythroid compartment showed a moderate anemia that remained stable throughout the life of the animals. After examining the etiology of this anemia, we found that it was caused by an impaired maturation of precursors within the bone marrow and spleen. There was an expansion of early erythroblasts, but these cells failed to efficiently mature. It was apparent that a failure to differentiate, termed ineffective erythropoiesis, occurred at the stage where cell cycle exit normally occurs. Using phenotypically stage-matched erythroid precursors from mutant animals and controls, we examined pathways that were either globally up- or downregulated to gain a better understanding of how this block in differentiation may occur and what the contribution of Rb was to the coupling of cell cycle exit to differentiation during erythropoiesis [96,97].

Consistent with the role of Rb in cell cycle exit, there was a failure to repress S-phase genes and particularly E2F transcription factor targets in the Rb-null erythrocytes. Surprisingly the most downregulated gene sets were all components of the mitochondrial electron transport chain and oxidative phosphorylation (OXPHOS) pathways. This result suggested a link between cell cycle regulation and mitochondrial biogenesis in erythroid cells. In agreement with the gene expression data there was both a reduced mitochondrial mass and mitochondrial DNA content in the Rb-null erythroid cells. There are numerous examples demonstrating how primary defects in mitochondrial function or biogenesis can lead to ineffective erythropoiesis in both humans [98-100] and experimental animals [101-103]. Our studies using lineage restricted gene deletion to allow *in vivo *studies allowed us to find a previously unappreciated link between cell cycle regulation and modulation of mitochondrial function during cellular differentiation, which appears critical at the mid-maturation erythroblast stage. Our initial work suggested that this phenomenon was likely to be mediated by modulation of the PPARγ-coactivator (PGC) transcriptional axis. In particular, we obtained some evidence to suggest that reduced expression of PGC-1β may play a role in this phenotype. Much work still remains to be done to further characterize the link that was observed between cell cycle regulation and mitochondrial biogenesis. Similar observations have been made in the context of proliferating fibroblasts, where it was suggested that the molecular control of this process occurs through modulation of transcription factors known to interact with the PGC family of coactivators [104].

The phenotype observed in the erythroid Rb-null mice closely resembled the ineffective erythropoiesis that is seen in human myelodysplastic syndrome (MDS). It is interesting to note that defects in mitochondrial structure and function have frequently been seen in the erythroid cells in MDS [98,99]. Concomitantly, cell cycle deregulation is thought to underlie the pathophysiology of MDS and the most frequently identified molecular defect in human MDS involves epigenetic silencing of the cell cycle inhibitory protein CDKN2B/p15INK4B [105,106]. Additionally, the anemia present in the Rb-null mice cannot be corrected even in the presence of high-level wild-type hematopoietic chimerism, suggesting that this may be the type of lesion that could allow a clonal disease like MDS to result in an anemia [105]. Our findings suggest a potential link between these observations that may lead to more effectively targeted therapies. If these features of MDS are linked together, then it may be useful to target both of these lesions simultaneously to strike at the "Achilles' heel" of MDS. The insights gained from this work may also suggest candidate genes that could be involved in the pathogenesis of MDS.

#### Outstanding questions regarding Rb and erythropoiesis

We have been able to uncover an interesting nexus between cell cycle exit during erythropoiesis and the regulation of mitochondrial biogenesis, which is mediated by Rb. This insight allows us to gain a greater understanding of normal erythropoiesis. Additionally, it is likely that this work will allow us to gain insight into the pathogenesis of MDS and possibly other disease states where ineffective erythropoiesis occurs. Importantly, while cell cycle exit is impaired in the Rb-null erythroid cells, cell cycle exit continues to occur to some extent in these cells and a significant proportion of the erythrocytes mature and are functional in the peripheral blood. It will be important to delineate the factors that are responsible for this continued ability to exit the cell cycle in the absence of Rb. Moreover, while the aforementioned studies have largely focused on homeostatic adult erythropoiesis, it will be important to delineate whether the "Rb pathway" functions differently during the extensive expansion in red cell mass that occurs in the course of ontogeny [107]. It will also be interesting to examine how the genes that play a role in promoting the increased proliferation during the early stages of erythropoiesis are coupled to differentiation. For example, it is likely that cyclins D2 and D3 are coupled to the differentiation of these progenitors to allow coordinated proliferation and maturation of these cells [15]. It is clear that alterations in the differentiation kinetics of RBC progenitors can impact how differentiation occurs, as exemplified by alterations in globin gene expression resulting from treatment with S-phase inhibitors ([81]. We only have a descriptive understanding of these phenomena currently, but it is likely that further molecular links similar to those we have described are playing a critical role here. Recent evidence from human genetic studies indicates that genes like c-Myb may have an important impact on the differentiation characteristics of these cells [108-110]. The further study of cell cycle regulation in the seemingly "well understood" differentiation model of erythropoiesis is likely to yield many new and fruitful insights into the general molecular networks that coordinate differentiation and proliferation.

## Conclusion

The use of lineage and temporally controlled somatic deletion strategies has allowed the development of complex *in vivo *models with which to study the roles of genes in both development and organ homeostasis. Questions previously unable to be studied regarding the role of Rb in the context of the adult HSCs and hematopoiesis can now begin to be addressed. Whilst helping to clarify previous ambiguity regarding the phenotype of loss of Rb, these studies have also revealed previously unrecognized roles for Rb in the regulation of HSCs and their microenvironment and in the regulation of mitochondrial function during terminal erythropoiesis.

## Competing interests

The authors declare that they have no competing interests.

## Authors' contributions

CRW, VGS and SHO drafted the manuscript. All authors read and approved the final manuscript.
